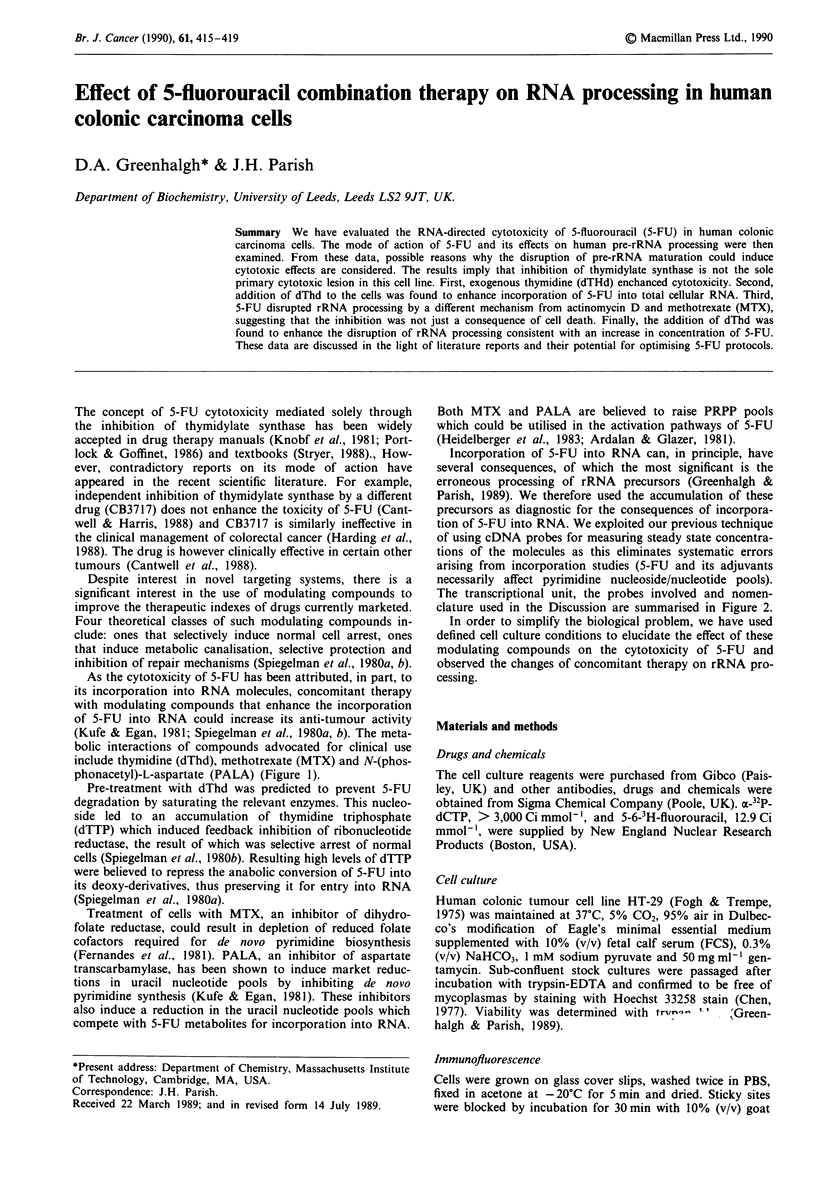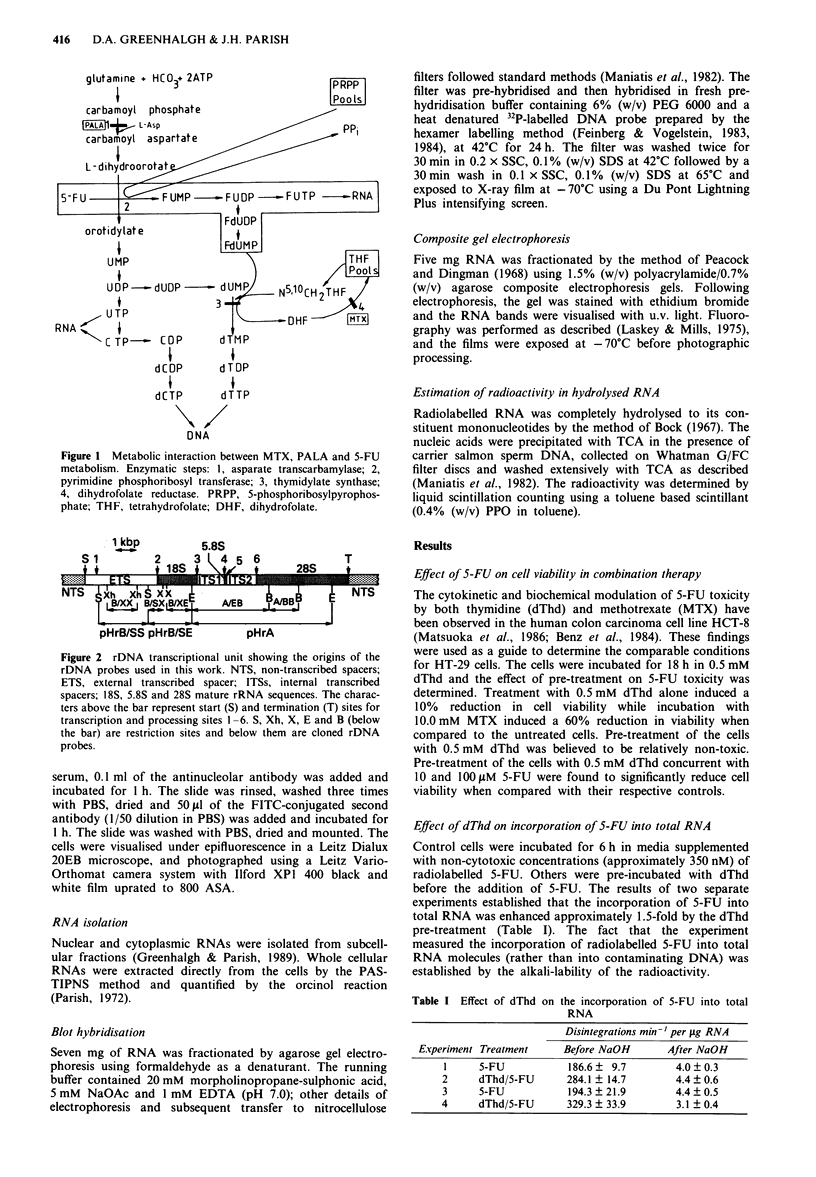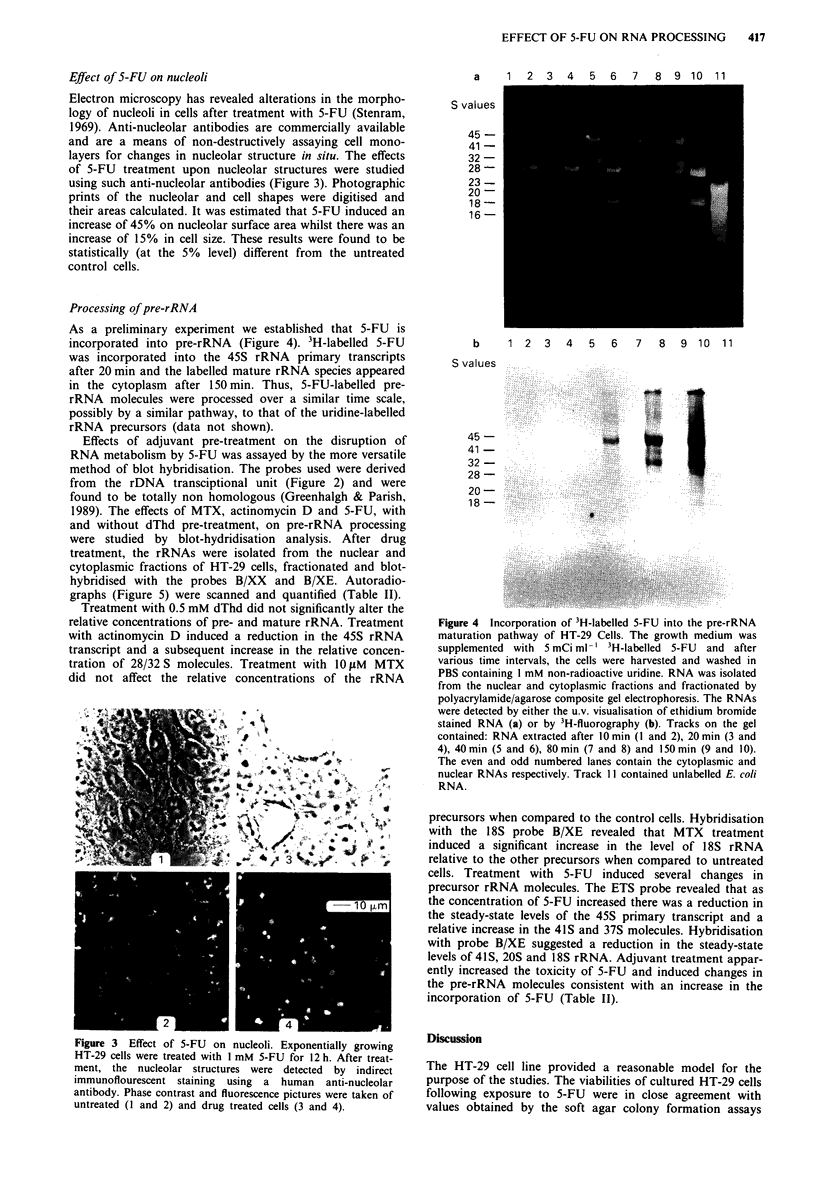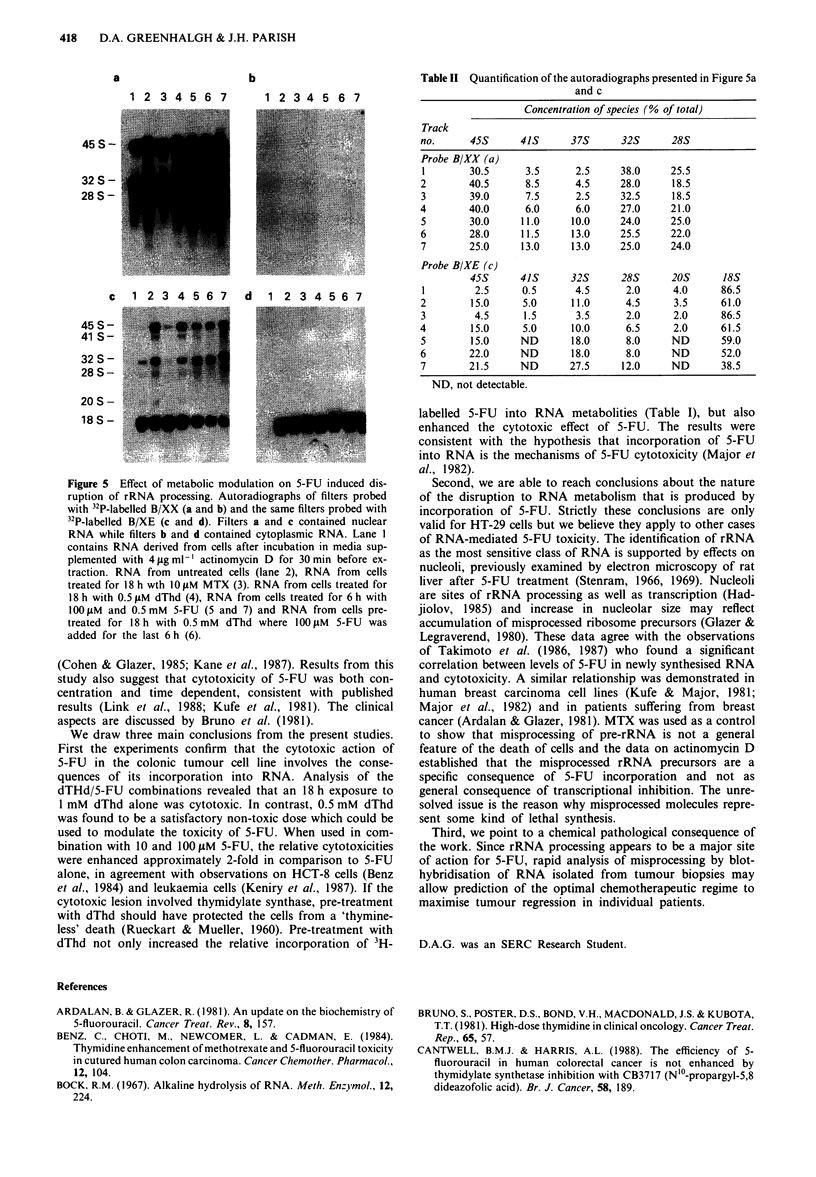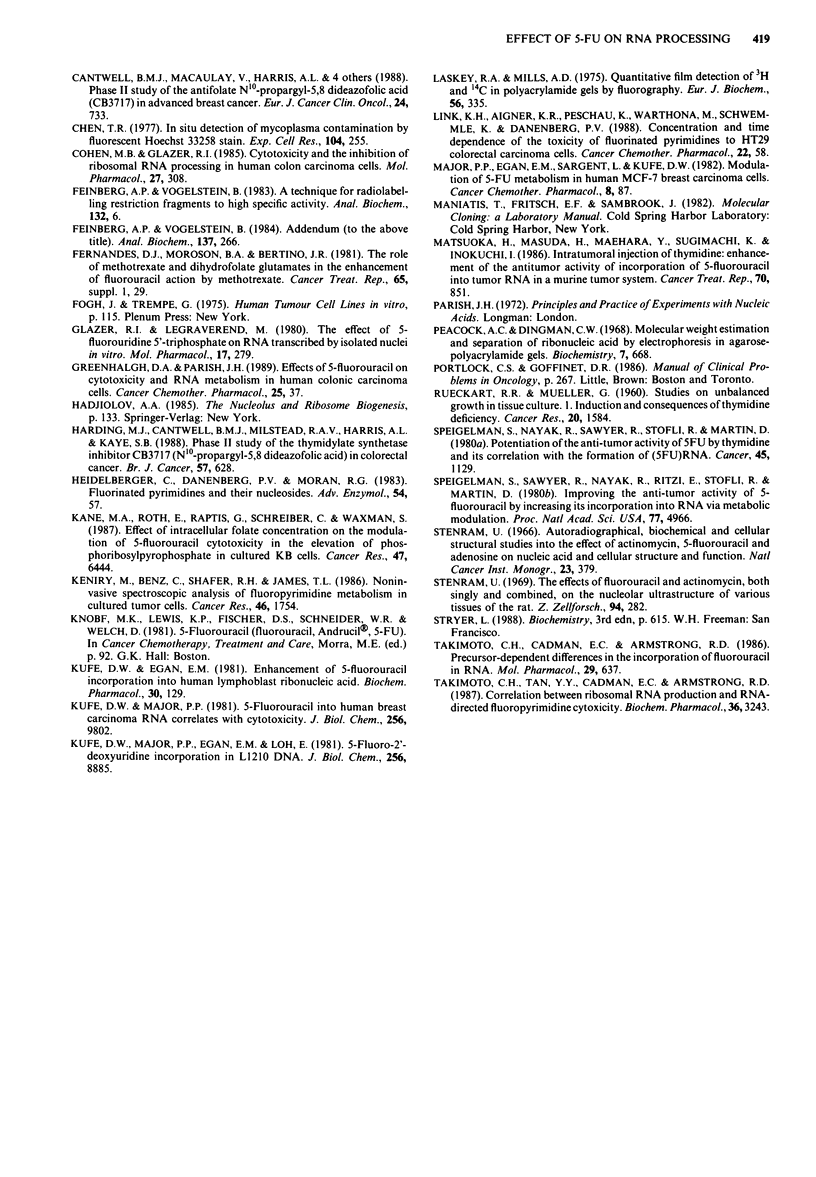# Effect of 5-fluorouracil combination therapy on RNA processing in human colonic carcinoma cells.

**DOI:** 10.1038/bjc.1990.91

**Published:** 1990-03

**Authors:** D. A. Greenhalgh, J. H. Parish

**Affiliations:** Department of Biochemistry, University of Leeds, U.K.

## Abstract

**Images:**


*Br. J. Cancer* (1990), **61**, 415–419 © Macmillan Press Ltd., 1990

### Effect of 5-fluorouracil combination therapy on RNA processing in human colonic carcinoma cells

D.A. Greenhalgh* & J.H. Parish

*Department of Biochemistry, University of Leeds, Leeds LS2 9JT, UK.*

**Summary** We have evaluated the RNA-directed cytotoxicity of 5-fluorouracil (5-FU) in human colonic carcinoma cells. The mode of action of 5-FU and its effects on human pre-rRNA processing were then examined. From these data, possible reasons why the disruption of pre-rRNA maturation could induce cytotoxic effects are considered. The results imply that inhibition of thymidylate synthase is not the sole primary cytotoxic lesion in this cell line. First, exogenous thymidine (dTHd) enchanced cytotoxicity. Second, addition of dThd to the cells was found to enhance incorporation of 5-FU into total cellular RNA. Third, 5-FU disrupted rRNA processing by a different mechanism from actinomycin D and methotrexate (MTX), suggesting that the inhibition was not just a consequence of cell death. Finally, the addition of dThd was found to enhance the disruption of rRNA processing consistent with an increase in concentration of 5-FU. These data are discussed in the light of literature reports and their potential for optimising 5-FU protocols.

The concept of 5-FU cytotoxicity mediated solely through the inhibition of thymidylate synthase has been widely accepted in drug therapy manuals (Knobf *et al.*, 1981; Portlock & Goffinet, 1986) and textbooks (Stryer, 1988)., However, contradictory reports on its mode of action have appeared in the recent scientific literature. For example, independent inhibition of thymidylate synthase by a different drug (CB3717) does not enhance the toxicity of 5-FU (Cantwell & Harris, 1988) and CB3717 is similarly ineffective in the clinical management of colorectal cancer (Harding *et al.*, 1988). The drug is however clinically effective in certain other tumours (Cantwell *et al.*, 1988).

Despite interest in novel targeting systems, there is a significant interest in the use of modulating compounds to improve the therapeutic indexes of drugs currently marketed. Four theoretical classes of such modulating compounds include: ones that selectively induce normal cell arrest, ones that induce metabolic canalisation, selective protection and inhibition of repair mechanisms (Spiegelman *et al.*, 1980*a*, *b*).

As the cytotoxicity of 5-FU has been attributed, in part, to its incorporation into RNA molecules, concomitant therapy with modulating compounds that enhance the incorporation of 5-FU into RNA could increase its anti-tumour activity (Kufe & Egan, 1981; Spiegelman *et al.*, 1980*a*, *b*). The metabolic interactions of compounds advocated for clinical use include thymidine (dThd), methotrexate (MTX) and *N*-(phosphonacetyl)-L-aspartate (PALA) (Figure 1).

Pre-treatment with dThd was predicted to prevent 5-FU degradation by saturating the relevant enzymes. This nucleoside led to an accumulation of thymidine triphosphate (dTTP) which induced feedback inhibition of ribonucleotide reductase, the result of which was selective arrest of normal cells (Spiegelman *et al.*, 1980*b*). Resulting high levels of dTTP were believed to repress the anabolic conversion of 5-FU into its deoxy-derivatives, thus preserving it for entry into RNA (Spiegelman *et al.*, 1980*a*).

Treatment of cells with MTX, an inhibitor of dihydrofolate reductase, could result in depletion of reduced folate cofactors required for *de novo* pyrimidine biosynthesis (Fernandes *et al.*, 1981). PALA, an inhibitor of aspartate transcarbamylase, has been shown to induce market reductions in uracil nucleotide pools by inhibiting *de novo* pyrimidine synthesis (Kufe & Egan, 1981). These inhibitors also induce a reduction in the uracil nucleotide pools which compete with 5-FU metabolites for incorporation into RNA.

Both MTX and PALA are believed to raise PRPP pools which could be utilised in the activation pathways of 5-FU (Heidelberger *et al.*, 1983; Ardalan & Glazer, 1981).

Incorporation of 5-FU into RNA can, in principle, have several consequences, of which the most significant is the erroneous processing of rRNA precursors (Greenhalgh & Parish, 1989). We therefore used the accumulation of these precursors as diagnostic for the consequences of incorporation of 5-FU into RNA. We exploited our previous technique of using cDNA probes for measuring steady state concentrations of the molecules as this eliminates systematic errors arising from incorporation studies (5-FU and its adjuvants necessarily affect pyrimidine nucleoside/nucleotide pools). The transcriptional unit, the probes involved and nomenclature used in the Discussion are summarised in Figure 2.

In order to simplify the biological problem, we have used defined cell culture conditions to elucidate the effect of these modulating compounds on the cytotoxicity of 5-FU and observed the changes of concomitant therapy on rRNA processing.

### Materials and methods

### *Drugs and chemicals*

The cell culture reagents were purchased from Gibco (Paisley, UK) and other antibodies, drugs and chemicals were obtained from Sigma Chemical Company (Poole, UK). $\alpha$-$^{32}$P-dCTP, > 3,000 Ci mmol$^{-1}$, and 5-6-$^{3}$H-fluorouracil, 12.9 Ci mmol$^{-1}$, were supplied by New England Nuclear Research Products (Boston, USA).

### *Cell culture*

Human colonic tumour cell line HT-29 (Fogh & Trempe, 1975) was maintained at 37°C, 5% $CO_2$, 95% air in Dulbecco's modification of Eagle's minimal essential medium supplemented with 10% (v/v) fetal calf serum (FCS), 0.3% (v/v) $NaHCO_3$, 1 mM sodium pyruvate and 50 mg ml$^{-1}$ gentamycin. Sub-confluent stock cultures were passaged after incubation with trypsin-EDTA and confirmed to be free of mycoplasmas by staining with Hoechst 33258 stain (Chen, 1977). Viability was determined with [illegible] (Greenhalgh & Parish, 1989).

### *Immunofluorescence*

Cells were grown on glass cover slips, washed twice in PBS, fixed in acetone at −20°C for 5 min and dried. Sticky sites were blocked by incubation for 30 min with 10% (v/v) goat

*Present address: Department of Chemistry, Massachusetts Institute of Technology, Cambridge, MA, USA.
Correspondence: J.H. Parish.
Received 22 March 1989; and in revised form 14 July 1989.

**Figure 1** Metabolic interaction between MTX, PALA and 5-FU metabolism. Enzymatic steps: 1, asparate transcarbamylase; 2, pyrimidine phosphoribosyl transferase; 3, thymidylate synthase; 4, dihydrofolate reductase. PRPP, 5-phosphoribosylpyrophosphate; THF, tetrahydrofolate; DHF, dihydrofolate.

**Figure 2** rDNA transcriptional unit showing the origins of the rDNA probes used in this work. NTS, non-transcribed spacers; ETS, external transcribed spacer; ITSs, internal transcribed spacers; 18S, 5.8S and 28S mature rRNA sequences. The characters above the bar represent start (S) and termination (T) sites for transcription and processing sites 1–6. S, Xh, X, E and B (below the bar) are restriction sites and below them are cloned rDNA probes.

serum, 0.1 ml of the antinucleolar antibody was added and incubated for 1 h. The slide was rinsed, washed three times with PBS, dried and 50 $\mu$l of the FITC-conjugated second antibody (1/50 dilution in PBS) was added and incubated for 1 h. The slide was washed with PBS, dried and mounted. The cells were visualised under epifluorescence in a Leitz Dialux 20EB microscope, and photographed using a Leitz Vario-Orthomat camera system with Ilford XP1 400 black and white film uprated to 800 ASA.

### *RNA isolation*

Nuclear and cytoplasmic RNAs were isolated from subcellular fractions (Greenhalgh & Parish, 1989). Whole cellular RNAs were extracted directly from the cells by the PAS-TIPNS method and quantified by the orcinol reaction (Parish, 1972).

### *Blot hybridisation*

Seven mg of RNA was fractionated by agarose gel electrophoresis using formaldehyde as a denaturant. The running buffer contained 20 mM morpholinopropane-sulphonic acid, 5 mM NaOAc and 1 mM EDTA (pH 7.0); other details of electrophoresis and subsequent transfer to nitrocellulose filters followed standard methods (Maniatis *et al.*, 1982). The filter was pre-hybridised and then hybridised in fresh pre-hydridisation buffer containing 6% (w/v) PEG 6000 and a heat denatured $^{32}$P-labelled DNA probe prepared by the hexamer labelling method (Feinberg & Vogelstein, 1983, 1984), at 42°C for 24 h. The filter was washed twice for 30 min in 0.2 × SSC, 0.1% (w/v) SDS at 42°C followed by a 30 min wash in 0.1 × SSC, 0.1% (w/v) SDS at 65°C and exposed to X-ray film at −70°C using a Du Pont Lightning Plus intensifying screen.

### *Composite gel electrophoresis*

Five mg RNA was fractionated by the method of Peacock and Dingman (1968) using 1.5% (w/v) polyacrylamide/0.7% (w/v) agarose composite electrophoresis gels. Following electrophoresis, the gel was stained with ethidium bromide and the RNA bands were visualised with u.v. light. Fluorography was performed as described (Laskey & Mills, 1975), and the films were exposed at −70°C before photographic processing.

### *Estimation of radioactivity in hydrolysed RNA*

Radiolabelled RNA was completely hydrolysed to its constituent mononucleotides by the method of Bock (1967). The nucleic acids were precipitated with TCA in the presence of carrier salmon sperm DNA, collected on Whatman G/FC filter discs and washed extensively with TCA as described (Maniatis *et al.*, 1982). The radioactivity was determined by liquid scintillation counting using a toluene based scintillant (0.4% (w/v) PPO in toluene).

### Results

### *Effect of 5-FU on cell viability in combination therapy*

The cytokinetic and biochemical modulation of 5-FU toxicity by both thymidine (dThd) and methotrexate (MTX) have been observed in the human colon carcinoma cell line HCT-8 (Matsuoka *et al.*, 1986; Benz *et al.*, 1984). These findings were used as a guide to determine the comparable conditions for HT-29 cells. The cells were incubated for 18 h in 0.5 mM dThd and the effect of pre-treatment on 5-FU toxicity was determined. Treatment with 0.5 mM dThd alone induced a 10% reduction in cell viability while incubation with 10.0 mM MTX induced a 60% reduction in viability when compared to the untreated cells. Pre-treatment of the cells with 0.5 mM dThd was believed to be relatively non-toxic. Pre-treatment of the cells with 0.5 mM dThd concurrent with 10 and 100 $\mu$M 5-FU were found to significantly reduce cell viability when compared with their respective controls.

### *Effect of dThd on incorporation of 5-FU into total RNA*

Control cells were incubated for 6 h in media supplemented with non-cytotoxic concentrations (approximately 350 nM) of radiolabelled 5-FU. Others were pre-incubated with dThd before the addition of 5-FU. The results of two separate experiments established that the incorporation of 5-FU into total RNA was enhanced approximately 1.5-fold by the dThd pre-treatment (Table I). The fact that the experiment measured the incorporation of radiolabelled 5-FU into total RNA molecules (rather than into contaminating DNA) was established by the alkali-lability of the radioactivity.

**Table I** Effect of dThd on the incorporation of 5-FU into total RNA

| | | *Disintegrations min$^{-1}$ per $\mu$g RNA* | |
|---|---|---|---|
| *Experiment* | *Treatment* | *Before NaOH* | *After NaOH* |
| 1 | 5-FU | 186.6 ± 9.7 | 4.0 ± 0.3 |
| 2 | dThd/5-FU | 284.1 ± 14.7 | 4.4 ± 0.6 |
| 3 | 5-FU | 194.3 ± 21.9 | 4.4 ± 0.5 |
| 4 | dThd/5-FU | 329.3 ± 33.9 | 3.1 ± 0.4 |

*Effect of 5-FU on nucleoli*

Electron microscopy has revealed alterations in the morphology of nucleoli in cells after treatment with 5-FU (Stenram, 1969). Anti-nucleolar antibodies are commercially available and are a means of non-destructively assaying cell monolayers for changes in nucleolar structure *in situ*. The effects of 5-FU treatment upon nucleolar structures were studied using such anti-nucleolar antibodies (Figure 3). Photographic prints of the nucleolar and cell shapes were digitised and their areas calculated. It was estimated that 5-FU induced an increase of 45% on nucleolar surface area whilst there was an increase of 15% in cell size. These results were found to be statistically (at the 5% level) different from the untreated control cells.

*Processing of pre-rRNA*

As a preliminary experiment we established that 5-FU is incorporated into pre-rRNA (Figure 4). $^3$H-labelled 5-FU was incorporated into the 45S rRNA primary transcripts after 20 min and the labelled mature rRNA species appeared in the cytoplasm after 150 min. Thus, 5-FU-labelled pre-rRNA molecules were processed over a similar time scale, possibly by a similar pathway, to that of the uridine-labelled rRNA precursors (data not shown).

Effects of adjuvant pre-treatment on the disruption of RNA metabolism by 5-FU was assayed by the more versatile method of blot hybridisation. The probes used were derived from the rDNA transciptional unit (Figure 2) and were found to be totally non homologous (Greenhalgh & Parish, 1989). The effects of MTX, actinomycin D and 5-FU, with and without dThd pre-treatment, on pre-rRNA processing were studied by blot-hydridisation analysis. After drug treatment, the rRNAs were isolated from the nuclear and cytoplasmic fractions of HT-29 cells, fractionated and blot-hybridised with the probes B/XX and B/XE. Autoradiographs (Figure 5) were scanned and quantified (Table II).

Treatment with 0.5 mM dThd did not significantly alter the relative concentrations of pre- and mature rRNA. Treatment with actinomycin D induced a reduction in the 45S rRNA transcript and a subsequent increase in the relative concentration of 28/32 S molecules. Treatment with 10 $\mu$M MTX did not affect the relative concentrations of the rRNA precursors when compared to the control cells. Hybridisation with the 18S probe B/XE revealed that MTX treatment induced a significant increase in the level of 18S rRNA relative to the other precursors when compared to untreated cells. Treatment with 5-FU induced several changes in precursor rRNA molecules. The ETS probe revealed that as the concentration of 5-FU increased there was a reduction in the steady-state levels of the 45S primary transcript and a relative increase in the 41S and 37S molecules. Hybridisation with probe B/XE suggested a reduction in the steady-state levels of 41S, 20S and 18S rRNA. Adjuvant treatment apparently increased the toxicity of 5-FU and induced changes in the pre-rRNA molecules consistent with an increase in the incorporation of 5-FU (Table II).

**Figure 3** Effect of 5-FU on nucleoli. Exponentially growing HT-29 cells were treated with 1 mM 5-FU for 12 h. After treatment, the nucleolar structures were detected by indirect immunoflourescent staining using a human anti-nucleolar antibody. Phase contrast and fluorescence pictures were taken of untreated (1 and 2) and drug treated cells (3 and 4).

**Figure 4** Incorporation of $^3$H-labelled 5-FU into the pre-rRNA maturation pathway of HT-29 Cells. The growth medium was supplemented with 5 mCi ml$^{-1}$ $^3$H-labelled 5-FU and after various time intervals, the cells were harvested and washed in PBS containing 1 mM non-radioactive uridine. RNA was isolated from the nuclear and cytoplasmic fractions and fractionated by polyacrylamide/agarose composite gel electrophoresis. The RNAs were detected by either the u.v. visualisation of ethidium bromide stained RNA (**a**) or by $^3$H-fluorography (**b**). Tracks on the gel contained: RNA extracted after 10 min (1 and 2), 20 min (3 and 4), 40 min (5 and 6), 80 min (7 and 8) and 150 min (9 and 10). The even and odd numbered lanes contain the cytoplasmic and nuclear RNAs respectively. Track 11 contained unlabelled *E. coli* RNA.

### Discussion

The HT-29 cell line provided a reasonable model for the purpose of the studies. The viabilities of cultured HT-29 cells following exposure to 5-FU were in close agreement with values obtained by the soft agar colony formation assays

**Figure 5** Effect of metabolic modulation on 5-FU induced disruption of rRNA processing. Autoradiographs of filters probed with $^{32}$P-labelled B/XX (**a** and **b**) and the same filters probed with $^{32}$P-labelled B/XE (**c** and **d**). Filters **a** and **c** contained nuclear RNA while filters **b** and **d** contained cytoplasmic RNA. Lane 1 contains RNA derived from cells after incubation in media supplemented with 4 $\mu$g ml$^{-1}$ actinomycin D for 30 min before extraction. RNA from untreated cells (lane 2), RNA from cells treated for 18 h wth 10 $\mu$M MTX (3). RNA from cells treated for 18 h with 0.5 $\mu$M dThd (4), RNA from cells treated for 6 h with 100 $\mu$M and 0.5 mM 5-FU (5 and 7) and RNA from cells pretreated for 18 h with 0.5 mM dThd where 100 $\mu$M 5-FU was added for the last 6 h (6).

(Cohen & Glazer, 1985; Kane *et al.*, 1987). Results from this study also suggest that cytotoxicity of 5-FU was both concentration and time dependent, consistent with published results (Link *et al.*, 1988; Kufe *et al.*, 1981). The clinical aspects are discussed by Bruno *et al.* (1981).

We draw three main conclusions from the present studies. First the experiments confirm that the cytotoxic action of 5-FU in the colonic tumour cell line involves the consequences of its incorporation into RNA. Analysis of the dTHd/5-FU combinations revealed that an 18 h exposure to 1 mM dThd alone was cytotoxic. In contrast, 0.5 mM dThd was found to be a satisfactory non-toxic dose which could be used to modulate the toxicity of 5-FU. When used in combination with 10 and 100 $\mu$M 5-FU, the relative cytotoxicities were enhanced approximately 2-fold in comparison to 5-FU alone, in agreement with observations on HCT-8 cells (Benz *et al.*, 1984) and leukaemia cells (Keniry *et al.*, 1987). If the cytotoxic lesion involved thymidylate synthase, pre-treatment with dThd should have protected the cells from a 'thymineless' death (Rueckart & Mueller, 1960). Pre-treatment with dThd not only increased the relative incorporation of $^3$H-labelled 5-FU into RNA metabolities (Table I), but also enhanced the cytotoxic effect of 5-FU. The results were consistent with the hypothesis that incorporation of 5-FU into RNA is the mechanisms of 5-FU cytotoxicity (Major *et al.*, 1982).

**Table II** Quantification of the autoradiographs presented in Figure 5a and c

| | *Concentration of species (% of total)* | | | | | |
|---|---|---|---|---|---|---|
| *Track no.* | *45S* | *41S* | *37S* | *32S* | *28S* | |
| *Probe B/XX (a)* | | | | | | |
| 1 | 30.5 | 3.5 | 2.5 | 38.0 | 25.5 | |
| 2 | 40.5 | 8.5 | 4.5 | 28.0 | 18.5 | |
| 3 | 39.0 | 7.5 | 2.5 | 32.5 | 18.5 | |
| 4 | 40.0 | 6.0 | 6.0 | 27.0 | 21.0 | |
| 5 | 30.0 | 11.0 | 10.0 | 24.0 | 25.0 | |
| 6 | 28.0 | 11.5 | 13.0 | 25.5 | 22.0 | |
| 7 | 25.0 | 13.0 | 13.0 | 25.0 | 24.0 | |
| *Probe B/XE (c)* | | | | | | |
| | *45S* | *41S* | *32S* | *28S* | *20S* | *18S* |
| 1 | 2.5 | 0.5 | 4.5 | 2.0 | 4.0 | 86.5 |
| 2 | 15.0 | 5.0 | 11.0 | 4.5 | 3.5 | 61.0 |
| 3 | 4.5 | 1.5 | 3.5 | 2.0 | 2.0 | 86.5 |
| 4 | 15.0 | 5.0 | 10.0 | 6.5 | 2.0 | 61.5 |
| 5 | 15.0 | ND | 18.0 | 8.0 | ND | 59.0 |
| 6 | 22.0 | ND | 18.0 | 8.0 | ND | 52.0 |
| 7 | 21.5 | ND | 27.5 | 12.0 | ND | 38.5 |

ND, not detectable.

Second, we are able to reach conclusions about the nature of the disruption to RNA metabolism that is produced by incorporation of 5-FU. Strictly these conclusions are only valid for HT-29 cells but we believe they apply to other cases of RNA-mediated 5-FU toxicity. The identification of rRNA as the most sensitive class of RNA is supported by effects on nucleoli, previously examined by electron microscopy of rat liver after 5-FU treatment (Stenram, 1966, 1969). Nucleoli are sites of rRNA processing as well as transcription (Hadjiolov, 1985) and increase in nucleolar size may reflect accumulation of misprocessed ribosome precursors (Glazer & Legraverend, 1980). These data agree with the observations of Takimoto *et al.* (1986, 1987) who found a significant correlation between levels of 5-FU in newly synthesised RNA and cytotoxicity. A similar relationship was demonstrated in human breast carcinoma cell lines (Kufe & Major, 1981; Major *et al.*, 1982) and in patients suffering from breast cancer (Ardalan & Glazer, 1981). MTX was used as a control to show that misprocessing of pre-rRNA is not a general feature of the death of cells and the data on actinomycin D established that the misprocessed rRNA precursors are a specific consequence of 5-FU incorporation and not as general consequence of transcriptional inhibition. The unresolved issue is the reason why misprocessed molecules represent some kind of lethal synthesis.

Third, we point to a chemical pathological consequence of the work. Since rRNA processing appears to be a major site of action for 5-FU, rapid analysis of misprocessing by blot-hybridisation of RNA isolated from tumour biopsies may allow prediction of the optimal chemotherapeutic regime to maximise tumour regression in individual patients.

D.A.G. was an SERC Research Student.

### References

ARDALAN, B. & GLAZER, R. (1981). An update on the biochemistry of 5-fluorouracil. *Cancer Treat. Rev.*, **8**, 157.

BENZ, C., CHOTI, M., NEWCOMER, L. & CADMAN, E. (1984). Thymidine enhancement of methotrexate and 5-fluorouracil toxicity in cutured human colon carcinoma. *Cancer Chemother. Pharmacol.*, **12**, 104.

BOCK, R.M. (1967). Alkaline hydrolysis of RNA. *Meth. Enzymol.*, **12**, 224.

BRUNO, S., POSTER, D.S., BOND, V.H., MACDONALD, J.S. & KUBOTA, T.T. (1981). High-dose thymidine in clinical oncology. *Cancer Treat. Rep.*, **65**, 57.

CANTWELL, B.M.J. & HARRIS, A.L. (1988). The efficiency of 5-fluorouracil in human colorectal cancer is not enhanced by thymidylate synthetase inhibition with CB3717 (N$^{10}$-propargyl-5,8 dideazofolic acid). *Br. J. Cancer*, **58**, 189.

CANTWELL, B.M.J., MACAULAY, V., HARRIS, A.L. & 4 others (1988). Phase II study of the antifolate $N^{10}$-propargyl-5,8 dideazofolic acid (CB3717) in advanced breast cancer. *Eur. J. Cancer Clin. Oncol.*, **24**, 733.

CHEN, T.R. (1977). In situ detection of mycoplasma contamination by fluorescent Hoechst 33258 stain. *Exp. Cell Res.*, **104**, 255.

COHEN, M.B. & GLAZER, R.I. (1985). Cytotoxicity and the inhibition of ribosomal RNA processing in human colon carcinoma cells. *Mol. Pharmacol.*, **27**, 308.

FEINBERG, A.P. & VOGELSTEIN, B. (1983). A technique for radiolabelling restriction fragments to high specific activity. *Anal. Biochem.*, **132**, 6.

FEINBERG, A.P. & VOGELSTEIN, B. (1984). Addendum (to the above title). *Anal. Biochem.*, **137**, 266.

FERNANDES, D.J., MOROSON, B.A. & BERTINO, J.R. (1981). The role of methotrexate and dihydrofolate glutamates in the enhancement of fluorouracil action by methotrexate. *Cancer Treat. Rep.*, **65**, suppl. 1, 29.

FOGH, J. & TREMPE, G. (1975). *Human Tumour Cell Lines in vitro*, p. 115. Plenum Press: New York.

GLAZER, R.I. & LEGRAVEREND, M. (1980). The effect of 5-fluorouridine 5'-triphosphate on RNA transcribed by isolated nuclei *in vitro*. *Mol. Pharmacol.*, **17**, 279.

GREENHALGH, D.A. & PARISH, J.H. (1989). Effects of 5-fluorouracil on cytotoxicity and RNA metabolism in human colonic carcinoma cells. *Cancer Chemother. Pharmacol.*, **25**, 37.

HADJIOLOV, A.A. (1985). *The Nucleolus and Ribosome Biogenesis*, p. 133. Springer-Verlag: New York.

HARDING, M.J., CANTWELL, B.M.J., MILSTEAD, R.A.V., HARRIS, A.L. & KAYE, S.B. (1988). Phase II study of the thymidylate synthetase inhibitor CB3717 ($N^{10}$-propargyl-5,8 dideazofolic acid) in colorectal cancer. *Br. J. Cancer*, **57**, 628.

HEIDELBERGER, C., DANENBERG, P.V. & MORAN, R.G. (1983). Fluorinated pyrimidines and their nucleosides. *Adv. Enzymol.*, **54**, 57.

KANE, M.A., ROTH, E., RAPTIS, G., SCHREIBER, C. & WAXMAN, S. (1987). Effect of intracellular folate concentration on the modulation of 5-fluorouracil cytotoxicity in the elevation of phosphoribosylpyrophosphate in cultured KB cells. *Cancer Res.*, **47**, 6444.

KENIRY, M., BENZ, C., SHAFER, R.H. & JAMES, T.L. (1986). Noninvasive spectroscopic analysis of fluoropyrimidine metabolism in cultured tumor cells. *Cancer Res.*, **46**, 1754.

KNOBF, M.K., LEWIS, K.P., FISCHER, D.S., SCHNEIDER, W.R. & WELCH, D. (1981). 5-Fluorouracil (fluorouracil, Andrucil®, 5-FU). In *Cancer Chemotherapy, Treatment and Care*, Morra, M.E. (ed.) p. 92. G.K. Hall: Boston.

KUFE, D.W. & EGAN, E.M. (1981). Enhancement of 5-fluorouracil incorporation into human lymphoblast ribonucleic acid. *Biochem. Pharmacol.*, **30**, 129.

KUFE, D.W. & MAJOR, P.P. (1981). 5-Fluorouracil into human breast carcinoma RNA correlates with cytotoxicity. *J. Biol. Chem.*, **256**, 9802.

KUFE, D.W., MAJOR, P.P., EGAN, E.M. & LOH, E. (1981). 5-Fluoro-2'-deoxyuridine incorporation in L1210 DNA. *J. Biol. Chem.*, **256**, 8885.

LASKEY, R.A. & MILLS, A.D. (1975). Quantitative film detection of $^{3}H$ and $^{14}C$ in polyacrylamide gels by fluorography. *Eur. J. Biochem.*, **56**, 335.

LINK, K.H., AIGNER, K.R., PESCHAU, K., WARTHONA, M., SCHWEMMLE, K. & DANENBERG, P.V. (1988). Concentration and time dependence of the toxicity of fluorinated pyrimidines to HT29 colorectal carcinoma cells. *Cancer Chemother. Pharmacol.*, **22**, 58.

MAJOR, P.P., EGAN, E.M., SARGENT, L. & KUFE, D.W. (1982). Modulation of 5-FU metabolism in human MCF-7 breast carcinoma cells. *Cancer Chemother. Pharmacol.*, **8**, 87.

MANIATIS, T., FRITSCH, E.F. & SAMBROOK, J. (1982). *Molecular Cloning: a Laboratory Manual.* Cold Spring Harbor Laboratory: Cold Spring Harbor, New York.

MATSUOKA, H., MASUDA, H., MAEHARA, Y., SUGIMACHI, K. & INOKUCHI, I. (1986). Intratumoral injection of thymidine: enhancement of the antitumor activity of incorporation of 5-fluorouracil into tumor RNA in a murine tumor system. *Cancer Treat. Rep.*, **70**, 851.

PARISH, J.H. (1972). *Principles and Practice of Experiments with Nucleic Acids.* Longman: London.

PEACOCK, A.C. & DINGMAN, C.W. (1968). Molecular weight estimation and separation of ribonucleic acid by electrophoresis in agarose-polyacrylamide gels. *Biochemistry*, **7**, 668.

PORTLOCK, C.S. & GOFFINET, D.R. (1986). *Manual of Clinical Problems in Oncology*, p. 267. Little, Brown: Boston and Toronto.

RUECKART, R.R. & MUELLER, G. (1960). Studies on unbalanced growth in tissue culture. 1. Induction and consequences of thymidine deficiency. *Cancer Res.*, **20**, 1584.

SPEIGELMAN, S., NAYAK, R., SAWYER, R., STOFLI, R. & MARTIN, D. (1980*a*). Potentiation of the anti-tumor activity of 5FU by thymidine and its correlation with the formation of (5FU)RNA. *Cancer*, **45**, 1129.

SPEIGELMAN, S., SAWYER, R., NAYAK, R., RITZI, E., STOFLI, R. & MARTIN, D. (1980*b*). Improving the anti-tumor activity of 5-fluorouracil by increasing its incorporation into RNA via metabolic modulation. *Proc. Natl Acad. Sci. USA*, **77**, 4966.

STENRAM, U. (1966). Autoradiographical, biochemical and cellular structural studies into the effect of actinomycin, 5-fluorouracil and adenosine on nucleic acid and cellular structure and function. *Natl Cancer Inst. Monogr.*, **23**, 379.

STENRAM, U. (1969). The effects of fluorouracil and actinomycin, both singly and combined, on the nucleolar ultrastructure of various tissues of the rat. *Z. Zellforsch.*, **94**, 282.

STRYER, L. (1988). *Biochemistry*, 3rd edn, p. 615. W.H. Freeman: San Francisco.

TAKIMOTO, C.H., CADMAN, E.C. & ARMSTRONG, R.D. (1986). Precursor-dependent differences in the incorporation of fluorouracil in RNA. *Mol. Pharmacol.*, **29**, 637.

TAKIMOTO, C.H., TAN, Y.Y., CADMAN, E.C. & ARMSTRONG, R.D. (1987). Correlation between ribosomal RNA production and RNA-directed fluoropyrimidine cytoxicity. *Biochem. Pharmacol.*, **36**, 3243.